# Ultra-deep and quantitative saliva proteome reveals dynamics of the oral microbiome

**DOI:** 10.1186/s13073-016-0293-0

**Published:** 2016-04-21

**Authors:** Niklas Grassl, Nils Alexander Kulak, Garwin Pichler, Philipp Emanuel Geyer, Jette Jung, Sören Schubert, Pavel Sinitcyn, Juergen Cox, Matthias Mann

**Affiliations:** Department of Proteomics and Signal Transduction, Max-Planck Institute of Biochemistry, Am Klopferspitz 18, D-82152 Martinsried, Germany; PreOmics GmbH, Am Klopferspitz 19, D-82152 Martinsried, Germany; Novo Nordisk Foundation Center for Protein Research, Faculty of Health and Medical Sciences, University of Copenhagen, Blegdamsvej 3B, DK-2200 Copenhagen, Denmark; Max von Pettenkofer-Institut für Hygiene und Medizinische Mikrobiologie, Marchioninistr. 17, D-81377 München, Germany; Computational Systems Biochemistry, Max-Planck Institute of Biochemistry, Am Klopferspitz 18, D-82152 Martinsried, Germany

## Abstract

**Background:**

The oral cavity is home to one of the most diverse microbial communities of the human body and a major entry portal for pathogens. Its homeostasis is maintained by saliva, which fulfills key functions including lubrication of food, pre-digestion, and bacterial defense. Consequently, disruptions in saliva secretion and changes in the oral microbiome contribute to conditions such as tooth decay and respiratory tract infections. Here we set out to quantitatively map the saliva proteome in great depth with a rapid and in-depth mass spectrometry-based proteomics workflow.

**Methods:**

We used recent improvements in mass spectrometry (MS)-based proteomics to develop a rapid workflow for mapping the saliva proteome quantitatively and at great depth. Standard clinical cotton swabs were used to collect saliva form eight healthy individuals at two different time points, allowing us to study inter-individual differences and interday changes of the saliva proteome. To accurately identify microbial proteins, we developed a method called “split by taxonomy id” that prevents peptides shared by humans and bacteria or between different bacterial phyla to contribute to protein identification.

**Results:**

Microgram protein amounts retrieved from cotton swabs resulted in more than 3700 quantified human proteins in 100-min gradients or 5500 proteins after simple fractionation. Remarkably, our measurements also quantified more than 2000 microbial proteins from 50 bacterial genera. Co-analysis of the proteomics results with next-generation sequencing data from the Human Microbiome Project as well as a comparison to MALDI-TOF mass spectrometry on microbial cultures revealed strong agreement. The oral microbiome differs between individuals and changes drastically upon eating and tooth brushing.

**Conclusion:**

Rapid shotgun and robust technology can now simultaneously characterize the human and microbiome contributions to the proteome of a body fluid and is therefore a valuable complement to genomic studies. This opens new frontiers for the study of host–pathogen interactions and clinical saliva diagnostics.

**Electronic supplementary material:**

The online version of this article (doi:10.1186/s13073-016-0293-0) contains supplementary material, which is available to authorized users.

## Background

Using saliva for the diagnosis of medical conditions would be particularly attractive because it can be collected non-invasively and economically [1], but the complexity of the oral cavity and the multiple entities contributing to its homeostasis make this challenging. In addition to the secretions of oral grands, saliva contains cells shed from the epithelium of the oral cavity and harbors the oral microbiome. Promising steps towards the establishment of saliva protein biomarkers have already been undertaken [2, 3]. However, these studies either only considered around 100 proteins with antibody-based assays or employed relatively low throughput mass spectrometry (MS)-based proteomics with extensive fractionation, which generally precluded quantification [4].

Further interest in saliva has recently been fueled by the discovery that the oral microbiome and the gut microbiome are the most diverse ones of the human body and that they correlate well with each other [5]. There is now compelling evidence for a link between the human microbiome and conditions such as obesity, allergies, and even autoimmune diseases like multiple sclerosis [6–8]. In addition, tooth decay and other diseases of the oral cavity are known to be caused by bacteria but turn out to be insufficiently explained by one species alone [9, 10]. Therefore, first metagenomics and then metaproteomics studies have already aimed to relate bacterial composition to caries incidence [10, 11]. However, reproducible identification and consistent quantification of bacteria remain challenging. Dynamic, quantitative studies would be of great help to uncover the functional connections between microbial communities and the prevalent pathologies of the oral cavity.

During the past few years, our laboratory has focused on simplifying and streamlining the proteomics workflow, with the aim of bringing the technology closer to clinical applications. Here we set out to characterize the saliva proteome at the greatest depth possible while still minimizing steps that could compromise quantification. We also developed a rapid single-run analysis workflow, starting from standard clinical cotton swabs and delivering results in a few hours, while retaining a quantification depth of thousands of proteins. This allowed us to investigate changes in the saliva proteome upon perturbation in a healthy cohort. We also analyzed inter-individual differences in the saliva proteome and quantitatively addressed the long-standing question of the degree to which the plasma and saliva proteomes are correlated. Finally, we asked if our in-depth workflow can characterize the oral microbiome and its dynamics and confirmed detected species by the established method of culturing followed by Matrix-assisted laser desorption/ionization time of flight mass spectrometry (MALDI-TOF MS) as well as data from next-generation sequencing projects.

## Methods

### Experimental design

We collected saliva at two different time points from four female and four male, healthy, non-smoking individuals aged 24 to 40 years with Caucasian backgrounds. All subjects were asymptomatic, did not take any drugs or antiseptics, visited the dentist regularly, and showed no signs of inflammation, bleeding, or infection as judged by a medical student (N.G.). The study was approved by the ethics committee of the Max Planck Society and all donors provided their written informed consent to participate in this study and to publish the acquired results. The first collection was immediately after waking, before eating, drinking, or tooth brushing. The second collection took place at 10 a.m., at least 30 min after the donors had eaten breakfast and brushed their teeth. In addition, we collected three samples immediately after one another from the same donor, processed them in parallel, and determined the reproducibility of our workflow. Because this showed very high reproducibility (mean *R*^2^ = 0.92, Additional file 1: Figure S3b), we did not perform technical replicates in this study but decided to use our measurement time for the analysis of several donors and proteome states.

### Protein digestion and peptide purification

Following collection, the swabs were transferred to an Eppendorf tube containing 200 μl of lysis buffer (1 % sodium dodecyl carbonate (v/v), 10 mM tris (2-carboxyethyl) phosphine, 40 mM 2-chloroacetamide, 100 mM Tris buffer pH 8.5), thoroughly squeezed against the inner wall of the Eppendorf tube, and removed. We reproducibly recovered more than 100 μg of protein in this way as estimated by the Bradford protein assay. Sample preparation followed essentially the in-StageTip protocol [12]. Briefly, a total of 20 μg of protein was digested by adding 0.4 μg trypsin and LysC to our lysis buffer and incubating for 60 min at 37 °C while shaking. Following this short digestion, we acidified the peptides to a final concentration of 1 % trifluoroacetic acid (TFA) and loaded them on an SDB-RPS StageTip [13]. The filter was then washed and peptides were finally eluted with 60 μl 80 % acetonitrile (ACN) (v/v) and 1 % ammonium (v/v), dried in a SpeedVac concentrator, and resuspended in A* buffer (2 % ACN (v/v), 0.1 % TFA (v/v), pH 2) to a concentration of 1 g/l.

### Single run and prefractionated liquid chromatography-MS measurement

To obtain a deep saliva proteome, we used basic reversed phase chromatography to fractionate our eight waking samples prior to liquid chromatography (LC)-MS measurement. Approximately 15 μg of peptides were separated in an 80-min gradient on a 20-cm, 75-μm inner diameter column that was in-house packed with ReproSil-Pur C_18_ beads (Dr. Maisch GmbH, Germany). Concatenated fractions [14, 15] were dried in the SpeedVac concentrator and resuspended in A* buffer to a concentration of 1 g/l. Both the fractionated and the single run samples were subjected to a 100-min chromatography gradient using an EASY-nLC 1000 ultra-high pressure system (Thermo Fisher Scientific) and an in-house-made 40-cm column of the type described above. The chromatography was on-line coupled to a Q Exactive HF mass spectrometer (Thermo Fisher Scientific) by applying a spray voltage of 2.2 kV. The MS scan resolution was set to 120,000 at m/z 200, the scan range to 300 to 1650 m/z, and the maximum injection time to 55 ms. The 15 most intense ions per MS scan were selected for higher-energy collisional dissociation (HCD) fragmentation with an isolation width of 1.5 m/z and were measured at a resolution of 30,000. Dynamic exclusion was used with an exclusion time of 30 s.

### Raw data processing of human proteins

The raw files were analyzed in MaxQuant [16] (version 1.5.3.15). We analyzed the single runs and the fractionated samples together in order to exploit the match between runs algorithm, which enables the identification of peptides that were not selected for fragmentation in one run by checking whether these peptides were sequenced in another run (the maximum time deviation was 30 s of the recalibrated retention times) [17]. We used the Andromeda search engine [18] to search the detected features against the human reference proteome from Uniprot (downloaded on 24 June 2015; 90.5 K sequences, 3.2 million unique peptides of which 0.64 million were seven amino acids or more in length) and a list of 247 potential contaminants [16]. Only tryptic peptides that were at least seven amino acids in length with up to two missed cleavages were considered. The initial allowed mass tolerance was set to 4.5 ppm at the MS level and 0.5 Da at the MS/MS level. We set N-acetylation of proteins’ N-termini (42.010565 Da) and oxidation of methionine (15.994915 Da) as variable modifications and carbamidomethylation of cysteine as a fixed modification (57.021464 Da). A false discovery rate (FDR) of 1 % was imposed for peptide-spectrum matches (PSMs) and protein identification using a target–decoy approach. Relative quantification was performed using the default parameters of the MaxLFQ algorithm [19] with the minimum ratio count set to 1.

### Data analysis of human proteins

The “proteinGroups.txt” file produced by MaxQuant was further analyzed in Perseus (version 1.5.2.12). Proteins from the reverse database, proteins only identified by site, and contaminants were removed. We decided to consider all keratin type I and II proteins contaminants because we could not exclude the possibility that their presence in our samples was due to skin desquamation. Proteins were ranked according to the mean label-free quantification (LFQ) intensities of the fractionated waking and the postprandial samples of all donors. We performed one-dimensional (1D) annotation enrichment of the resulting logarithmized LFQ distribution for Gene Ontology (GO) terms and Uniprot keywords with a Benjamini–Hochberg FDR cutoff of 2 % as described [20]. For the comparison of plasma and saliva proteomes, we used triplicate plasma proteomes of two of our saliva donors measured with 45-min HPLC gradients [21]. These six raw files were processed together with the single run saliva files from the two donors using the MaxQuant settings from above. Principal component analysis (PCA) was done on the logarithmized LFQ intensities of all 16 single shot runs. The differences between the waking and postprandial proteomes were analyzed by filtering the list of quantified proteins for 100 % valid values in all 16 single run analyses and performing a two sided *t*-test on the logarithmized LFQ intensities with a Benjamini–Hochberg FDR cutoff of 5 % and the s0 parameter set to 0.1. We determined whether the significantly upregulated proteins at waking were enriched for certain Uniprot keywords compared with the entire proteome using a Fisher exact test with 2 % permutation-based FDR. The analogous analysis was performed for the significantly upregulated postprandial proteins.

### Raw data processing of human and bacterial proteins

For the analysis of human and bacterial proteins, we downloaded the fasta files of all named species of the human oral microbiome database [22] with more than five protein sequences (downloaded 24 June 2015; 1118.9 K bacterial protein sequences in total). Together with the human sequences the resulting database contained 1209.4 K protein sequences which correspond to 58.6 million unique peptides after in silico digestion and 5.9 million peptides seven amino acids or more in length, which we considered in our MaxQuant settings. Search parameters were essentially identical to the raw file processing of human proteins alone, except that we applied the split by taxonomy feature on the phylum level and only used unique peptides for quantification. Due to the split by taxonomy on the phylum level, peptides that are part of human and bacterial proteins or peptides that occur in proteins from two different phyla are neglected for protein identification. This, as well as using only unique peptides rather than razor peptides for quantification, guarantees that peptides shared by different phyla are not attributed to the wrong organism.

### Data analysis of the oral microbiome

For creating the taxonomic tree in Fig. 4, we determined the number of peptides that uniquely belonged to one species of our database and wrote this number above the respective edge of the genus. Peptides shared by certain genera were added to the number of the lowest taxonomy edge shared by these genera (Operating Taxonomy Unit). For Fig. 4 we excluded all genera that did not have at least one unique peptide. We extended the analysis for streptococci down to the species level. Bacterial genus abundance was estimated by adding the ten peptides of highest intensity per genus in analogy to the protein quantification in [23, 24]. Genera with less than ten peptides were excluded from quantification.

### Co-analysis with whole genome sequencing data from the human microbiome project

To compare our data with results obtained from whole genome sequencing (WGS), protein multifasta (PEP) was downloaded from the Human Microbiome Project (HMP) [25]. Fractionated and single run raw files were analyzed with the MaxQuant settings described above against the human reference proteome from Uniprot and the fasta file from HMP (3.8 million protein sequences, 127.3 million unique peptides). From the genomic side we downloaded 764 fastq files from the HMP (release of 2012) and trimmed them using Trimmomatic [26] (we removed adapter as well as leading and trailing sequences with quality lower than 10 Phred quality score; we also did not accept reads for further analysis with lengths less than 36 nucleotides) and aligned using BWA with default parameters [27]. A PCA of the reads per genus of the WGS dataset together with the top ten peptide intensities per genus across the median of all samples from MaxQuant was performed after Z-score scaling within each sample (Fig. 5d). We combined the body sites “saliva”, “tongue dorsum”, “attached keratinized gingiva”, “palatine tonsils” and “throat” from the HMP for our definition of mouth because these sites clustered tightly in a PCA. Furthermore, we performed hierarchical clustering (Euclidean distance coupled with Ward’s agglomeration method was used) on the resulting dataset and visualized the genus abundance per sample in a heatmap (using the R package *heatmap.2*) (Additional file 1: Figure S1).

### Microbiological processing of the samples

Together with the cotton swab collection after waking, all donors also collected whole saliva by passive drooling into a sterile tube. Samples were processed immediately after collection as follows. One Columbia and one chocolate blood agar plate for the aerobic and two Schaedler agar plates for the anaerobic culture were plated out with 50 μl saliva each. Aerobic cultures were incubated for 3 days at 37 °C and 5.8 % CO_2_. Anaerobic cultures were grown under anaerobic conditions at 37 °C for a minimum of 5 days. Plates were evaluated visually and all morphologically different colonies were subcultured for identification by MALDI-TOF MS.

### Identification by MALDI-TOF MS

Samples were measured in duplicates according to the standard protocol recommended by the manufacturer. In brief, a thin layer of bacteria taken from a single colony was smeared onto a polished steel target and overlaid with 1 μl of matrix solution containing 10 mg/ml of α-cyano-4-hydroxy-cinnamic acid in 50 % acetonitrile/2.5 % TFA (α-HCCA portioned matrix, Bruker Daltonik GmbH, Bremen, Germany). For measurements, a Microflex LT benchtop instrument operated by flexControl 3.3 software (Bruker Daltonik GmbH, Germany) was used. Spectra were acquired in the linear positive ion mode at a laser frequency of 60 Hz within a mass range of 2 to 20 kDa. The acceleration voltage was 20 kV, the IS2 voltage was maintained at 18.6 kV, and the extraction delay time was 200 ns. For data analysis, spectra were matched with the Bruker Taxonomy database version 4.0.0.1.

## Results and discussion

### In-depth quantification of the saliva proteome

**Fig. 1:**
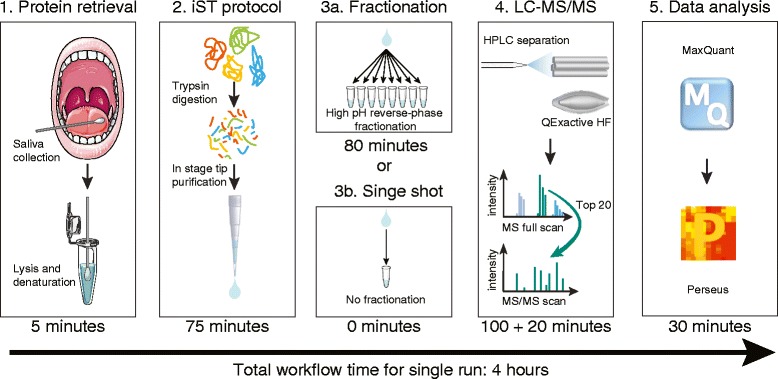
Workflow for ultra-deep and quantitative saliva proteomics. (1, 2) Saliva is collected with a sterile cotton swab and its proteins are denatured, digested, and purified according to the iST protocol [12]. (3) Depending on the desired proteome depth, samples are either separated into eight fractions or directly measured in single runs. (4) Peptides are measured by liquid chromatography–tandem mass spectrometry (LC-MS/MS). (5) MaxQuant identifies and quantifies the proteins and enables statistical analysis in the Perseus software environment. The required time for each of the steps is indicated below each panel

We obtained saliva from four male and four female healthy individuals using sterile cotton swabs as is done in routine clinical practice (Fig. 1, “Methods”). Donors were required to abstain from eating and drinking for at least 30 min prior to the collection to avoid food-based contamination or dilution effects. They were instructed to wipe the vestibule of the oral cavity, followed by the teeth and the sublingual compartment. Around 200 μg of total protein was recovered from each swab, an ample amount for repeated measurement using our recently developed in-StageTip digestion procedure [12]. Following an immediate digestion for one hour and purification, the resulting peptides were separated into eight fractions with basic reversed-phase chromatography [14, 15]. Each fraction as well as unfractionated sample was measured with a 100-min LC gradient on a Q Exactive HF mass spectrometer [28, 29]. Data were analyzed using the MaxQuant environment [16, 19].

**Fig. 2:**
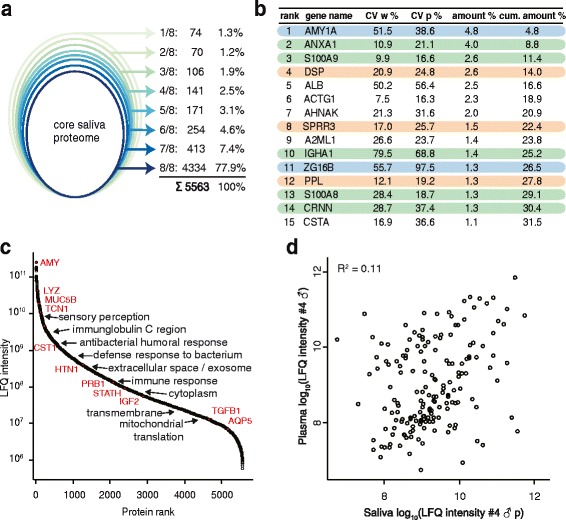
Deep human saliva proteomes of eight healthy donors. **a** *Ovals* represent the number of saliva proteins shared by the respective number of donors. The *outer oval* contains all proteins that were detected in at least one donor, whereas the inner oval contains all proteins found in each sample—the core proteome. The *numbers* on the *right* indicate the numbers of proteins exactly found in one donor, in two donors, and so on. **b** Gene names of the 15 most abundant saliva proteins, their coefficients of variation (CVs) across eight donors at waking (*w*) and after breakfast and tooth brushing (*p*), as well as their abundances in percentage of the total proteome and the cumulative protein abundances (*cum. amount*). The proteins in *blue* are digestive proteins, the proteins in *green* are part of immune defense, and the proteins in *red* are of epithelial origin. **c** Dynamic range plot of the saliva proteome with some key proteins in saliva highlighted in *red*. Significantly enriched GO terms or Uniprot keywords in specific abundance regions as determined by 1D annotation are listed. **d** Scatter plot of the LFQ intensities of the saliva proteome and the plasma proteome

Across our eight donors we identified more than 54,000 sequence-unique peptides and more than 5500 proteins, both at a false discovery rate (FDR) of 1 %. A total of 78 % of these proteins were detected in each donor, 90 % in at least six of eight donors, and only 1.3 % were unique to single donors (Fig. 2a). Thus, our sample collection protocol is robust and allows comparison of thousands of saliva proteins across individuals. For an individual donor, we identified a remarkable 5213 human proteins in the eight fractions—to our knowledge the deepest body fluid proteome recorded from an individual to date (Additional file 1: Figure S2a). To investigate the reasons for this extensive coverage, we inspected the MS signal of the most abundant proteins. Unlike other body fluids, the 15 most abundant proteins in saliva make up only 32 % of the total proteome mass (Fig. 2b), whereas in plasma and urine they already account for more than 90 % and 58 % of the total, respectively [30, 31].

The abundance ranked plot of the entire measured saliva proteome spans a dynamic range of six orders of magnitude of estimated absolute abundance (Fig. 2c). To bioinformatically investigate the saliva proteome as a function of abundance, we used 1D annotation enrichment in the Perseus environment for GO terms and Uniprot keywords [20]. “Antibacterial humoral response” and “defense response to bacterium” scored in the upper part of the abundance distribution (Fig. 2c). “Extracellular space” and “Extracellular exosome” were significant near the median, indicating that proteins making up this category are somewhat less abundant than most of the functional saliva proteins. The terms in the lowest abundance range included typical intracellular terms such as “cytoplasm” and “mitochondrial translation”.

There is an ongoing debate as to the extend that easily obtainable saliva could be used to measure plasma biomarkers by proxy [32]. We measured the plasma proteomes of two of our saliva donors in singe-run triplicate measurements [21] and compared them with the single-run saliva proteomes of the same donors. Due to the dynamic range challenges, fewer proteins were identified in plasma but more than 50 % of these were also identified in saliva. A scatter plot of the label-free quantification (LFQ) intensities of the proteins [19] that were identified in both body fluids reveals little correlation between these values (*R*^2^ = 0.11; Fig. 2d). Over the two individuals and all replicates, it was never higher than *R*^2^ = 0.20. We also considered the possibility that particular saliva components might show a higher correlation with the plasma proteome and collected one saliva sample from the opening of the duct of the parotid gland, one from the opening of the sublingual and submandibular gland, and one from gingiva. All these saliva proteomes revealed *R*^2^ values below 0.1 (Additional file 1: Figure S3). Thus, we conclude that the plasma and saliva proteomes show little overall correlation and that saliva cannot directly be used as a substitute for the determination of plasma protein levels.

To make our saliva results available to the community in a user-friendly format, we uploaded them to the MaxQB database [33]. For each protein of interest, a query will reveal whether it is present in our saliva proteome, its abundance rank, estimated absolute abundance, and other protein level information (Additional file 1: Figure S2b). Additionally, peptide evidence leading to protein identification as well as high-resolution precursor–fragment relationships are available for constructing targeted assays. The protein illustrated in Additional file 1: Figure S2b is transcobalamin-1 (TCN1), which is known to be secreted by the salivary glands and to protect cobalamin or vitamin B12 against acidity of the stomach. In addition, TCN1 functions as a transport protein in the blood, carrying excess cobalamin to the liver for storage. Cobalamin deficiency occurs in 20 % of individuals over the age of 60 years [34] and causes anemia, demyelinating disease, or both [35]. Due to cobalamin’s clinical significance, the physiological levels of TCN1 in blood have been characterized extensively in dedicated studies [36, 37], whereas here its levels are determined in the context of our system-wide investigation of thousands of other saliva proteins.

### A deep single-run workflow

The high proteome coverage achieved using fractionation motivated us to determine how much of the saliva proteome could be retrieved in a single-run or “single-shot” experiment [17]. We used the same 100-min gradients as before and measured saliva proteomes from the eight individuals mentioned above, each at two different time points, once immediately after waking before tooth brushing and once post-prandial after tooth brushing. Remarkably, an average of 3835 proteins could be identified and almost all of them (94 %) were also quantifiable (Additional file 1: Figure S4a). The results from three swabs taken at nearly the same time and processed independently but equally were highly similar with a mean coefficient of determination *R*^2^ of 0.92 (Additional file 1: Figure S4b). The difference between individuals was somewhat higher, with an *R*^2^ of 0.89, indicating that biological differences between individuals can also be captured by single-run measurements. Plotting the CVs for saliva proteome variation between the individuals showed that they did not primarily depend on protein abundance (Additional file 1: Figure S4c). This suggests that single-run analysis should be able to determine biological differences across a wide abundance range. As the single-shot proteome still quantifies more than 3700 proteins, which include nearly all the functional categories described above, very rapid and medium throughput characterization of saliva may be possible in the clinic.

### Dynamics of the saliva proteome in a cohort

The oral cavity is subject to a variety of conditions in daily life. Despite several studies investigating, for instance, changing cortisol levels [38], to our knowledge intraday changes in the saliva proteome have not yet been investigated in depth.

**Fig. 3:**
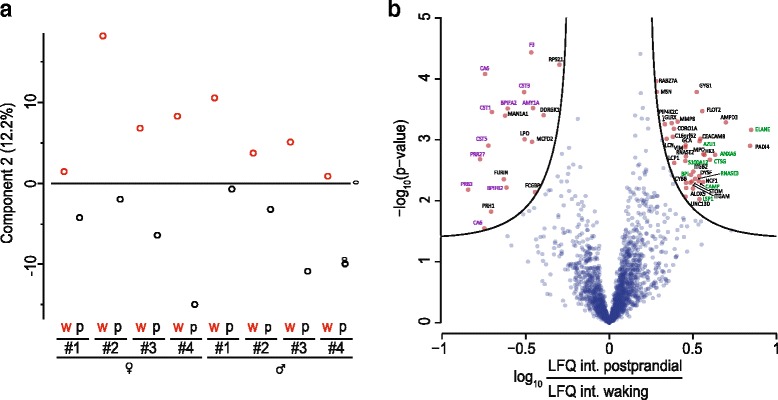
Intraday dynamics of the human saliva proteome. **a** PCA of the 16 saliva samples showing that component 2 separates samples based on the collection time (*w* = waking and *p* = postprandial). **b** Differentially regulated proteins between w and p as determined by plotting the *t*-test significance (5 % permutation-based FDR) versus the logarithmized fold change of LFQ intensity (*volcano plot*). Protein data points are labeled by their gene names. The *green gene names* indicate genes with the Uniprot keyword “antibiotic” or “antimicrobial”, the *purple gene names* indicate proteins with the Uniprot keyword “secreted”

To uncover dynamic changes, we first performed a principal component analysis (PCA) on all 16 single-run proteomes. Component 1 of the PCA separated weakly by sex (Additional file 1: Figure S5), whereas component 2 separated the two proteome states (waking versus post-prandial after tooth brushing) and this difference was even more pronounced when inspected on a person-by-person basis (Fig. 3a). To determine the proteins responsible for the PCA clustering, we filtered for 100 % valid LFQ values and plotted significance (5 % FDR) versus fold change (Fig. 3b). The proteins that were significantly upregulated at waking were enriched in the keywords “antibiotic” (*p* = 7.7 × 10^−9^, enrichment factor (ef) = 33) and “antimicrobial” (*p* = 6.6 × 10^−8^, ef = 24). The proteins with significantly higher abundance in the postprandial state were enriched for the terms “thiol protease inhibitor” and “secreted” (*p* = 3.3 × 10^−5^, ef = 42, and *p* = 8.7 × 10^−9^, ef = 6, respectively). Serving as a positive control, levels of alpha amylase (AMY1A), a protein that initiates the breakdown of complex oligosaccharides, were consistently upregulated after the meal. Thus, the shifts in protein abundance between our two measurement time points demonstrate that MS-based proteomics can now robustly capture biologically meaningful dynamic changes in body fluid proteomes.

### Identification of bacterial proteomes in human saliva

Due to the prominent role of the oral microbiome in health and disease, we investigated whether we could detect bacterial species in the deep saliva proteomes. For this purpose, we downloaded the complete Uniprot protein sequences of all named oral bacterial species that had been identified by 16S rRNA sequencing in a recent study [22]. The resulting database was about 11 times larger than the human one alone.

**Fig. 4:**
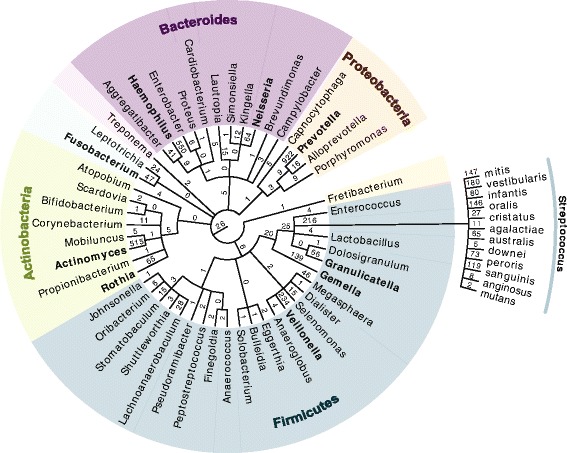
Taxonomy tree of 50 bacterial genera with evidence at the peptide level. The number of peptides that were specifically attributable to this position on the taxonomic tree are given above the *edges* of the graph. Genera in *bold* were also detected after bacterial culture of the saliva samples followed by MALDI-TOF MS measurement. For the genus *Streptococcus* the tree is extended down to the species level

In metaproteomics it is not straightforward to assign peptides to bacterial phyla because some amino acid sequences are part of proteins from different phyla. We addressed this issue by applying the “split by taxonomy” feature in MaxQuant, which avoids the formation of protein groups between different phyla. Together with the exclusive use of unique peptides for protein quantification, this functionality prevents the same peptide from contributing to the identification and quantification of proteins in different phyla (“Methods”). Split by taxonomy id is, therefore, relevant only for protein identification but not for peptide identification or quantification. However, bacteria in the oral cavity can have substantial sequence identity (Additional file 1: Figure S6a, b) [39]. As closely related bacteria share many sequences, one therefore needs to find the most appropriate taxonomy rank for applying the split by taxonomy id. To address this question, we placed identified bacterial peptides on a taxonomic tree such that the number of shared peptides is noted on each branch (Fig. 4). These shared peptides do not allow discrimination of the branches below. Split by taxonomy at a certain taxonomic rank prevents peptides shared at the ranks above from contributing to the identification of proteins. As in the case of human and microbial proteins above, this prevents the misassignment of peptides to phyla from which they do not necessarily originate. Placing the split at the phylum level turned out to be a good compromise between use of peptides for identification and quantification on the one hand and stringency of identification of bacteria on the other hand (Additional file 1: Figure S6) and we used this setting for all following analyses.

**Fig. 5:**
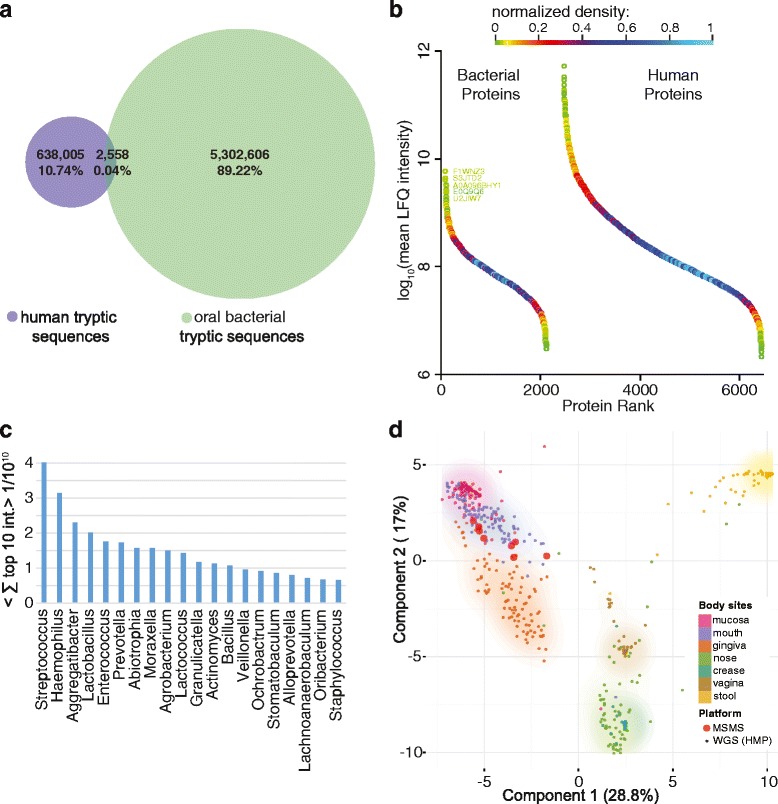
Quantitative distribution of bacterial proteins. **a** Number of nonredundant tryptic sequences considered in our MaxQuant search space for human (*violet*) and oral (*green*) bacteria. The percentage of shared peptides between human and bacteria among all nonredundant peptides in our search is 0.04 %. **b** Dynamic range plot of the saliva proteome searched against a combined human and bacterial sequence database. The protein density is color coded and the names of the most abundant proteins are given. **c** The sum of the top ten peptide intensities per genus serves as a quantitative measure of genus abundance. The 20 most abundant genera are depicted. **d** PCA of whole genome sequencing (*WGS*) data from the human microbiome project (*HMP*) co-analyzed with our saliva proteome data (*MSMS*). The MS-based proteomics data (*MSMS*) tightly co-localizes with the mouth sites from the human microbiome project

The presence of bacteria in the oral cavity also raises the question of whether proteins from them might considerably impair the human protein quantification presented above. To address this question we determined the nonredundant tryptic peptides that were seven or more amino acids long in our human and our oral bacteria database, which is the minimum length considered in our analysis. Among these tryptic peptides, the percentage of peptides with identical sequences between humans and bacteria was only 0.043 % (Fig. 5a). Hence, the quantification bias of human proteins due to bacteria is marginal. This analysis also indicates that bacterial contamination of mammalian proteome samples does not impair protein quantification considerably as long as only peptides of seven amino acids or more in length are considered.

Similarly, ingested proteins from food could, in principle, be erroneously assigned to human or bacterial proteins. To estimate the magnitude of these effects, we performed an analogous analysis on bovine and wheat as representative parts of a Western breakfast diet and determined the number of sequence identical peptides to humans and bacteria (Additional file 1: Figure S7). Except for bovine and human the percentage of overlapping peptide sequences is far below 1 %. Due to an overlap of 20.7 % among the considered human and bovine peptides, our in silico analysis does not exclude the possibility of quantification bias. However, proteins that substantially differ between waking and the postprandial state in Fig. 3 do not include proteins from human milk or human muscle, as would be expected if these differences were due to a bovine diet.

Remarkably, a search of our deep saliva proteome data sets using our standard, stringent search criteria (1 % FDR at the peptide and protein levels) resulted in the identification of 2234 different bacterial proteins. In total, we found evidence for 50 different bacterial genera from nine different phyla. This represents 50 % of the named genera identified by next-generation sequencing with corresponding, annotated UniProt proteomes and therefore present in our database. The proteomic coverage of bacterial genera is remarkably high given the restricted database and the modest measuring time. The distribution of peptides specific for particular genera was highly unequal, ranging from only 1 to 1069 for the genus *Streptococcus*, for which Fig. 4 shows a detailed taxonomic tree down to the species level. At least 12 different such *Streptococcus* species were present in our deep saliva proteome. The most abundant species was *Streptococcus mitis*, but we also detected peptides unique to *Streptococcus mutans*, a main contributor to dental caries formation.

Standard MALDI-TOF MS as now routinely used in clinical microbiology found evidence of 14 different genera in our saliva samples, with an average of six genera per donor (“Methods”). In each case, shotgun proteomics had also identified the genus in the same sample without the need to cultivate the bacteria prior to processing. A rough comparison with the number of MS-identified peptides for genera identified by MALDI-TOF MS suggests that they were generally the more abundant ones (Fig. 4). While the goal in clinical microbiology is to identify the presence of one or a few pathogens responsible for an infection, rather than a total inventory of the microbiome, it is nevertheless notable that unbiased and relatively straightforward shotgun proteomics of saliva identified these bacteria without intervening cultivation directly from a cotton swab. This identification would presumably have been much easier still in the case of a dominating pathogen.

### The quantitative oral metaproteome

To further investigate the unexpectedly large number of bacterial protein identifications, we plotted their cumulative percentage as a function of abundance rank (Additional file 1: Figure S8). Among the first 1000 proteins only 5 % were bacterial proteins. This proportion increased steadily until it reached 35 % for the total set of about 6000 proteins. Expressed as the percentage of bacterial proteins per 100 proteins, the chance to identify bacterial proteins reached more than 50 % towards the limit of detection. This suggests that increasing the depth of proteomic analysis would preferentially uncover further bacterial proteins and that our coverage of the oral metaproteome is far from saturation. As the depth of our bacterial detection increases in the future, it may also be possible to analyze bacterial pathways and how they change across different conditions of the oral cavity.

The simultaneous detection of bacterial and human proteomes in our samples allowed us to directly compare them quantitatively (Fig. 5b). The most abundant bacterial protein was F1WNZ3, the *Moraxella catarrhalis* homolog of chaperone protein HscA, which is involved in maturation of iron-sulfur-containing proteins. Its abundance was only 100-fold lower than the top human protein, alpha-amylase 1. Further highly abundant proteins of the bacterial metaproteome included proteins with household functions, such as A0A096BHY1, which is a glyceraldehyde-3-phosphate dehydrogenase, or E0Q9Q6, a subunit of DNA polymerase III. Sequence alignment in Perseus showed that many of the very abundant bacterial proteins were highly conserved. Therefore, peptides from different species likely contribute to their abundance.

The number of significantly identified human proteins decreased to about 4000 in the combined search space (Fig. 5b). Thus, almost a third of the overall protein count of 6197 is due to the microbiome. The bacterial proteins originated from four main phyla, with 300 to 800 uniquely assigned proteins, each of which spanned the entire abundance range (Additional file 1: Figure S9). In analogy to the top-three-peptide method commonly used in label-free abundance estimation of proteins [23, 24], we defined an approximate quantitative measure of the abundance of a bacterial genus as the summed MS intensity of the top ten most abundant peptides across all samples. These data were available for nearly all genera and, as in the protein case, comparing just the ten highest peptide intensities should be a better measure than summing all peptides, which would tend to overestimate abundance differences. The top ten peptides were determined among all peptides of a genus, not just unique peptides. This comes at the disadvantage that peptides shared by two genera could lead to an overestimation of the taxon’s abundance. Considering only unique peptides would have put genera with large sequence identity at a great disadvantage compared with genera with relatively distinct peptide sequences. However, this shows that adequate quantification of bacterial genera by their proteomes is challenging and at the present coverage our quantitative readouts should be considered as approximations rather than exact quantifications.

We applied our bacterial quantification measure to all detected genera and plotted the abundance of the top 20 (Fig. 5c). As expected from quantification performed by 16S RNA sequencing [40, 41], *Streptococcus* was the most abundant genus. The top ten genera did not show drastic differences in abundance (the integrated MS peptide signal of the top ten peptides was 4.0 × 10^10^ for *Streptococcus* and 1.4 × 10^10^ for *Lactococcus*). While we believe that the quantitative trends between bacteria are correct, more accurate quantification would require deeper sequence coverage of the bacterial proteomes.

The Human Microbiome Project (HMP) has generated large datasets of human microbiomes using next-generation sequencing [25]. We compared our quantitative bacterial proteomes with the whole genome sequencing data of the HMP in a PCA (Fig. 5d) and a heatmap of genera against samples (Additional file 1: Figure S1). The different body sites clustered separately in the genome data, with our proteomic data strikingly co-localizing with the oral microbiome. We did not expect such close co-localization given that both datasets originate from different samples and individuals. However, these results are in agreement with previous findings showing that the oral microbiome has relatively low diversity among individuals (beta diversity) [25]. The human microbiome study had collected samples from different locations in the mouth, but these data cluster together in the PCA, suggesting that the microbiome is similar throughout the oral cavity.

### Variation and dynamics of the metaproteome

**Fig. 6:**
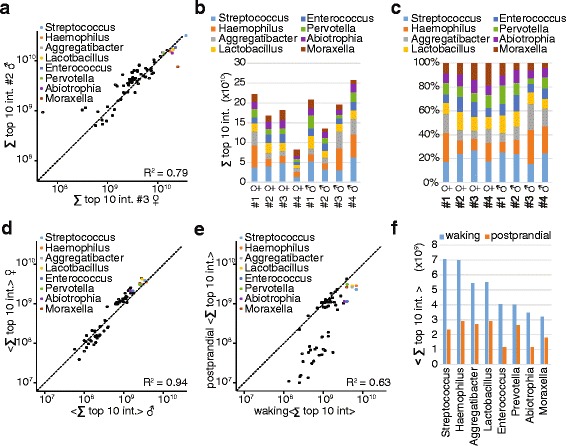
Bacterial composition across donors and time points. **a** Typical scatter plot for two donors of the bacterial genera quantified by the sum of their top ten peptide intensities from fractionated proteome measurements. The eight most abundant genera are color coded. **b** Absolute quantification of the eight most abundant bacterial species depicted for each individual donor and (**c**) normalized to 100 %. **d** Mean genus quantities between males and females treated as groups from single run measurements. **e** Comparison of mean bacterial abundances at waking to the mean bacterial abundances in the postprandial state. **f** Reduction in bacterial abundance between the waking saliva samples and the postprandial samples

Apart from estimating bacterial abundances, our data allow a quantitative comparison of the same genus upon perturbation or across individuals. Overall, individuals varied little in their bacterial diversity in accordance with the HMP [25]. A scatterplot of two typical donors reveals that bacterial abundances are similar for many of them, with a strong mean *R*^2^ of 0.82 (Fig. 6a shows a typical scatter plot). However, there are also genera that varied up to tenfold.

The cumulative abundances of the top eight bacterial genera across all donors indicate differences in total bacterial mass of up to threefold (Fig. 6b). Variation in the relative abundance of genera is much smaller (Fig. 6c) and the same analysis at the level of the five most abundant phyla showed similar variation.

When aggregating males and females separately, the two groups exhibited very comparable bacterial abundances that were highly correlated (*R*^2^ = 0.94; Fig. 6d). Thus, proteomics indicates that sex differences in the oral microbiome are minor. In contrast, bacterial abundance changed drastically after eating breakfast and tooth brushing. The high abundance bacterial genera were reduced 2.5-fold on average, while the lower abundant ones generally showed even stronger reduction (Fig. 6e, f). The *Streptococcus* genus, which contains *S. mutans*, was reduced by almost threefold after tooth brushing (Fig. 6f). It has been established that the *S. mutans* species is not the only one involved in cavity formation [42] and it would now be interesting to study the effects of different oral hygiene regimes on the oral bacterial community at the proteome level.

Our deep saliva proteomes also allow combined analysis of the human and bacterial proteome changes in response to the same perturbations. For instance, at waking, when bacterial abundance is high, the human saliva proteome was primed towards bacterial defense with substantial enrichment of proteins annotated with the Uniprot keywords “antibiotic” and “anti-microbioal”. Given the higher abundance of the microbiome at waking, this likely reflects the body’s effort to limit bacterial proliferation during the night when these populations are relatively undisturbed. This example illustrates the utility of the simultaneous detection of the human and bacterial proteomes for the study of the interplay of the host and microbiome.

## Conclusions

Here we employed shotgun proteomics with a state of the art workflow and identified more than 5500 proteins, the largest number of human proteins in a body fluid to date. Comparison with the plasma proteome established that the quantitative protein levels do not correlate.

We showed that shotgun proteomics can now readily determine 50 bacterial genera in saliva but the sequence coverage of bacterial proteins and organisms suggests that we have only scratched the surface of the oral bacterial proteome. Quantitative comparison to next-generation sequencing data from the HMP [25] revealed excellent agreement, suggesting that proteomics could provide a valuable complement to sequencing-based measurements of the human microbiome. Furthermore, proteomics appears uniquely positioned to study the interplay of the human immune system with commensurate and pathogenic bacteria on the protein level. With improving technology, our workflow might even become attractive for clinical microbiology since bacteria do not need to be grown and rapid bacterial resistance testing could become possible by directly measuring proteins that confer resistance to antibiotics. An important task for the future is to better characterize and annotate bacterial sequences in order to provide comprehensive, non-redundant databases for bacterial proteomics.

In conclusion, the depth and relatively straightforward nature of our workflow should make it a powerful new tool in the detection of biomarkers of diseases of the oral cavity as well as facilitate complementary studies of the microbiome in different contexts. In particular, proteomics appears uniquely positioned to study the interplay of the human immune system with commensurate and pathogenic bacteria at the systems level. We hope that such approaches will help to open new avenues in clinical application and for microbiology in the future.

## Availability of supporting data

The data sets supporting the results of this article are available in the proteomeXchange repository (http://www.proteomexchange.org), accession number PXD003028.

## Additional file

Additional file 1: Supplementary **Figures S1**-**S9**. (PDF 13519 kb)
