# Supplementary material for: Ultra-deep and quantitative saliva proteome reveals dynamics of the oral microbiome
Source: Genome Med. 2016 Apr 21;8:44. doi: 10.1186/s13073-016-0293-0 (PMC4841045; doi:10.1186/s13073-016-0293-0)
Supplement: Additional file 1: — Supplementary Figures S1-S9. (PDF 13519 kb) [file 13073_2016_293_MOESM1_ESM.pdf]

Figure S1

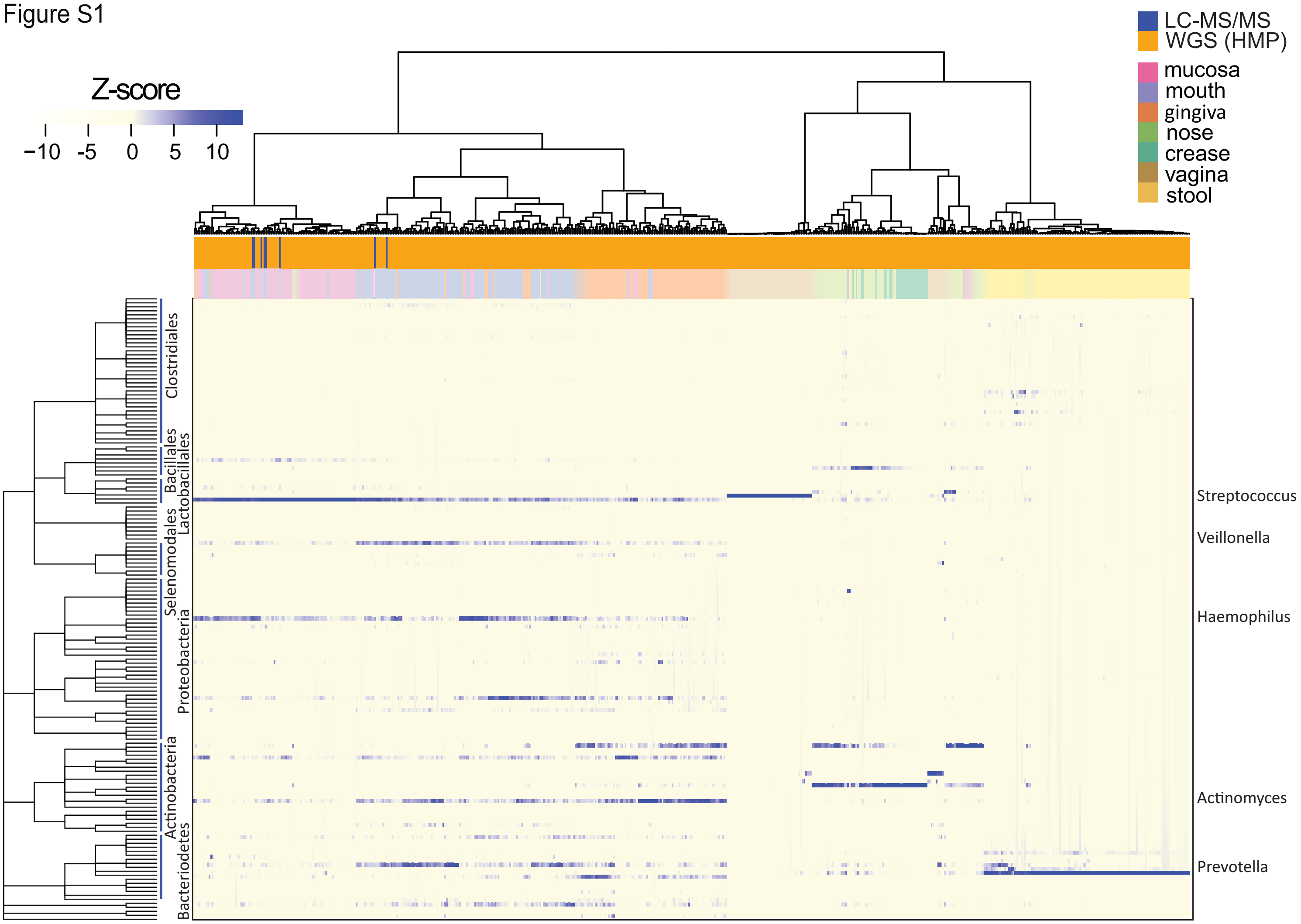

Figure S1: Comparison of species abundance between NGS and LC-MS/MS data across body sites. The dominant genus for different body sites stand out. The hierarchical clustering at the top reveals close colocalization of LC-MS/MS oral microbiome data with the oral microbiome obtained by NGS.

Figure S2

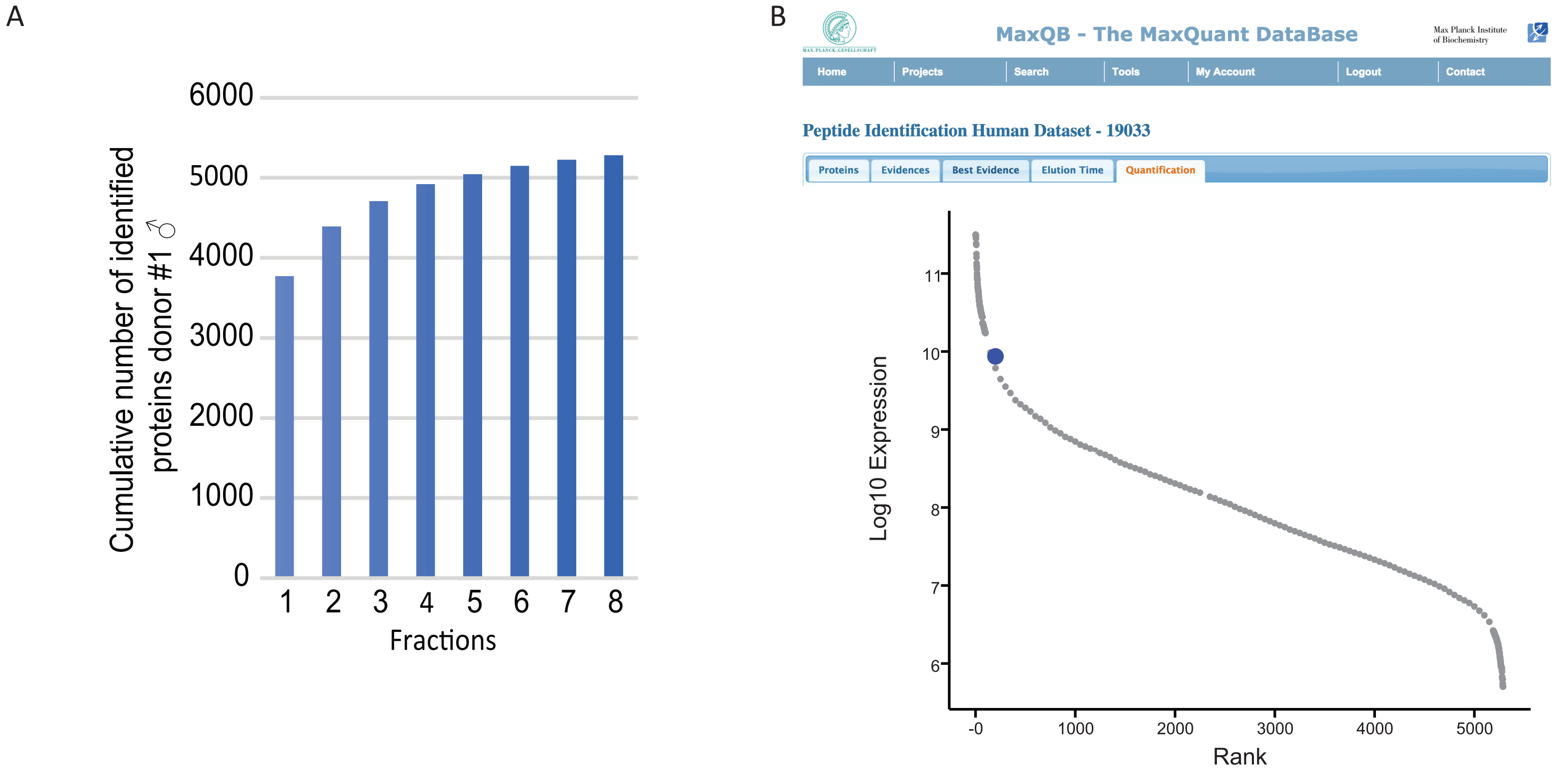

Figure S2: Contribution of fractionation and MaxQB

(A) Cumulative number of identified human saliva proteins for donor # 1 ♂ across eight basic reverse phase fractions. (B) Screenshot from our saliva dataset on MaxQB, a publicly accessible, user-friendly database for large scale MS projects. The LFQ intensity of Transcobalamin 1, an important transporter of Vitamin B12, was selected for closer inspection.

Figure S3

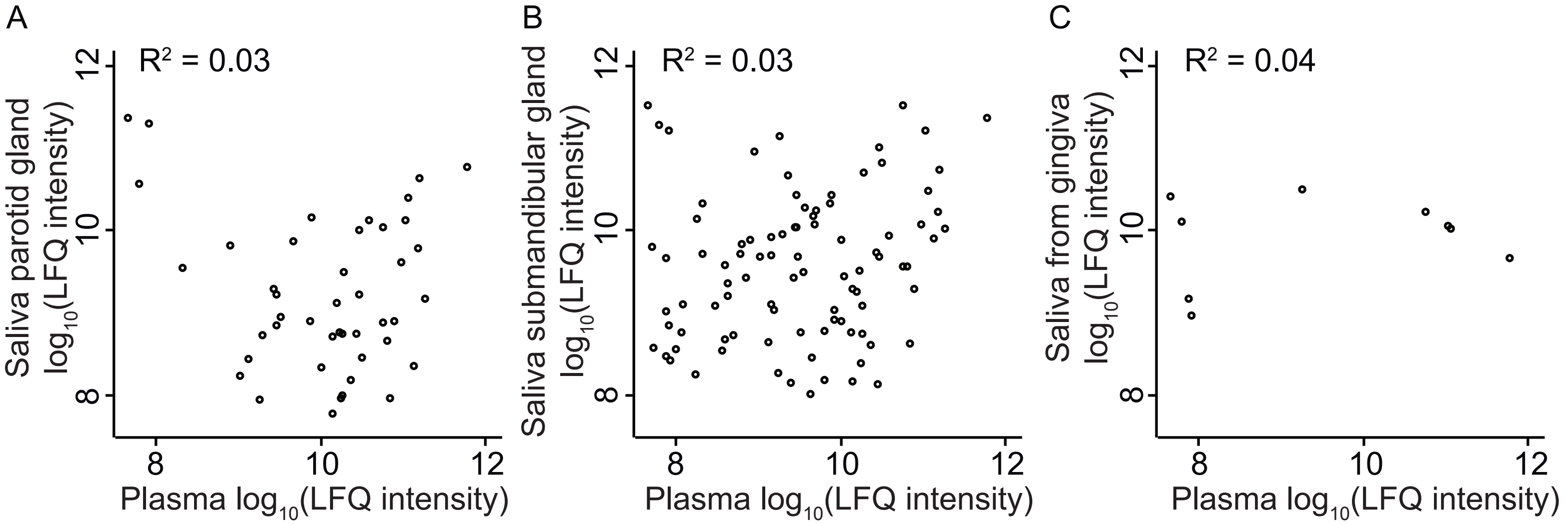

Figure S3: Comparison of the plasma proteome to saliva proteomes collected at specific sites

Scatter plots the plasma proteome and saliva proteomes with saliva collected from the opening of the duct of the parotid gland (A), the opening of the duct of the submandibular and sublingual gland (B) and saliva from gingiva (C).

Figure S4

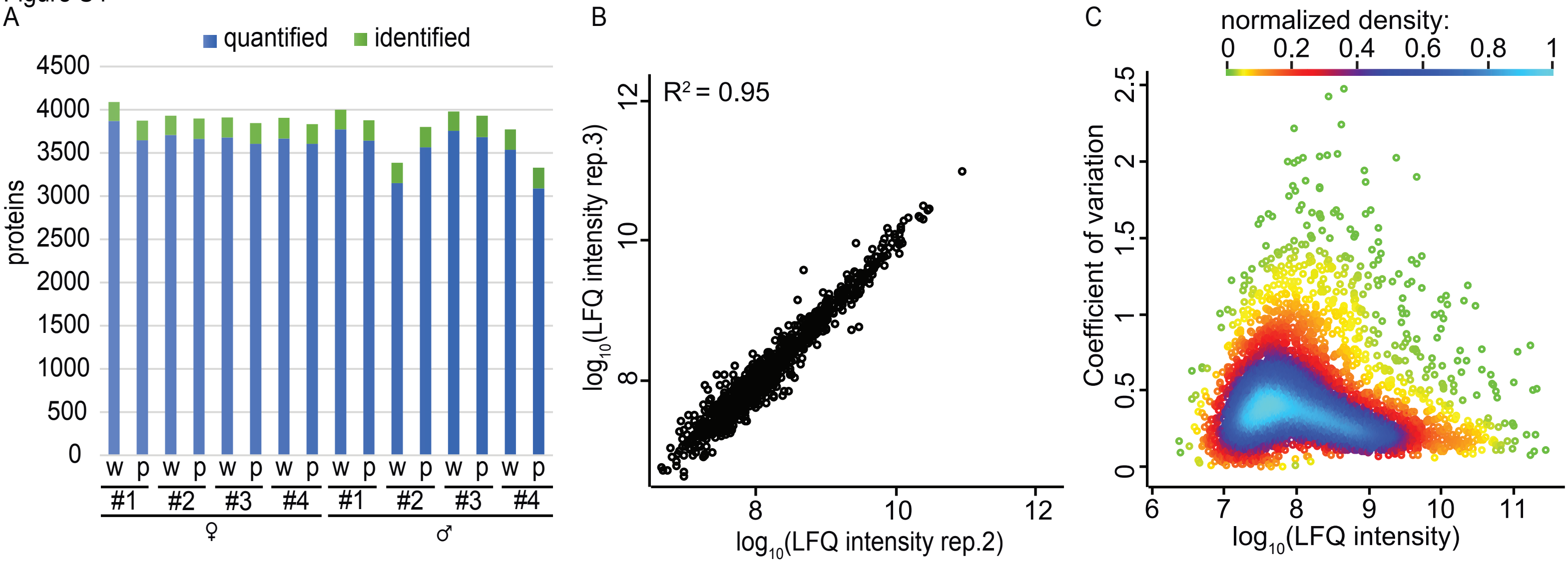

Figure S4: Single shot measurements of eight donors at two time points

(A) Number of quantified or only identified proteins form single run measurements of the waking (w) and postprandial (p) saliva samples of our eight donors. (B) Reproducibility of the protein quantification for two workflow replicates. (C) Coefficient of variation of LFQ protein intensities of our 16 single run measurements of eight donors plotted against protein abundance.

Figure S5

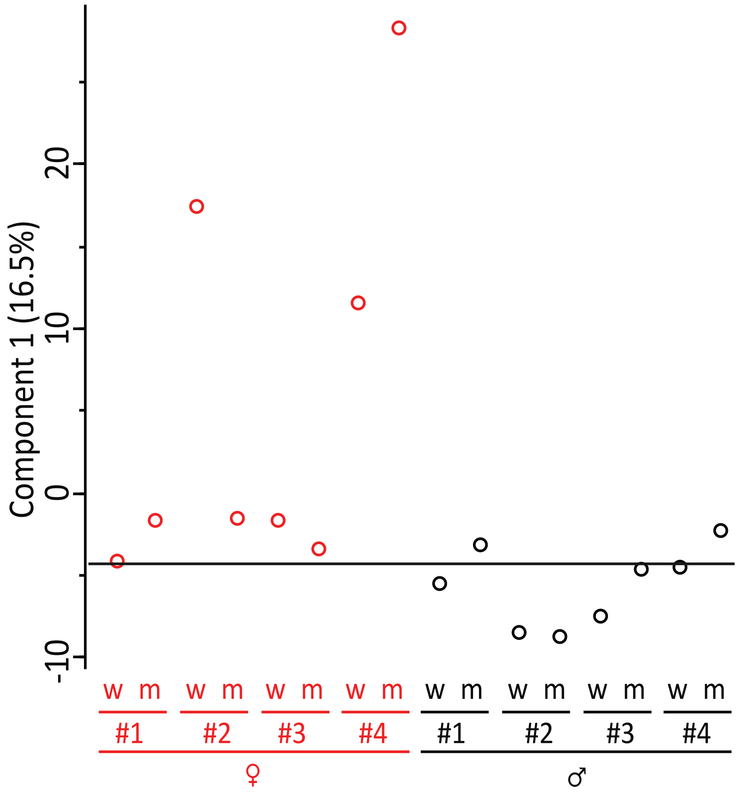

Figure S5: Principal component analysis of 16 single run proteomes weakly separates sexes
Principal component analysis (PCA) of the 16 saliva samples showing that component 1 has the tendency to separate samples based on sex (red = female donors, black = male donors).

Figure S6

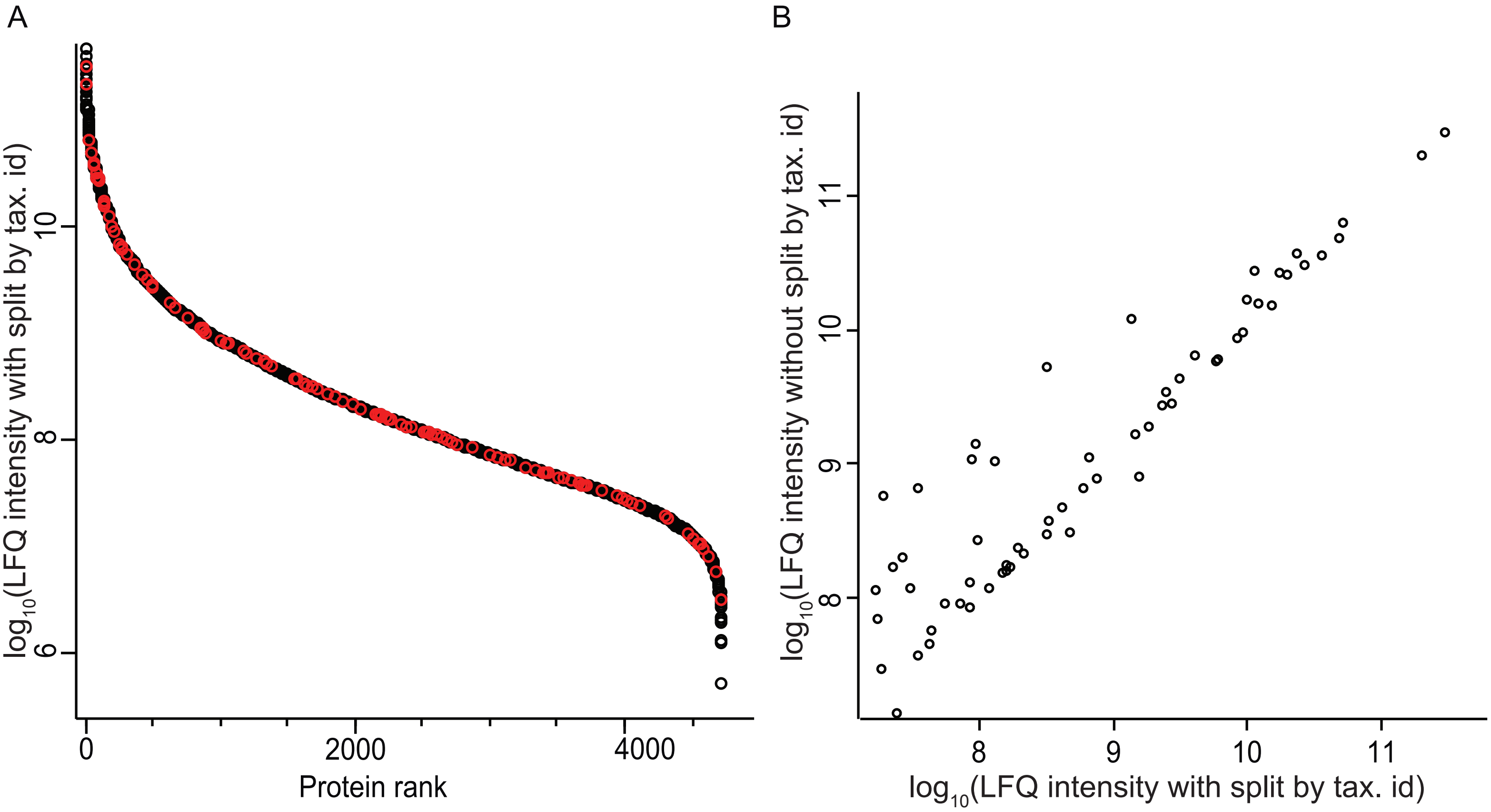

Figure S6: Comparison of split by taxonomy quantification to the plain MaxLFQ algorithm

(A) Distribution of all detected 163 proteins (red) with shared peptide sequences between different phyla or bacteria and humans in the protein abundance plot for donor #4 ♂. (B) Scatter plot of the LFQ intensities of the red proteins in A comparing the plain MaxLFQ algorithm to the split by taxonomy algorithm. Without using split by taxonomy id the LFQ values of certain proteins are overestimated.

Figure S7

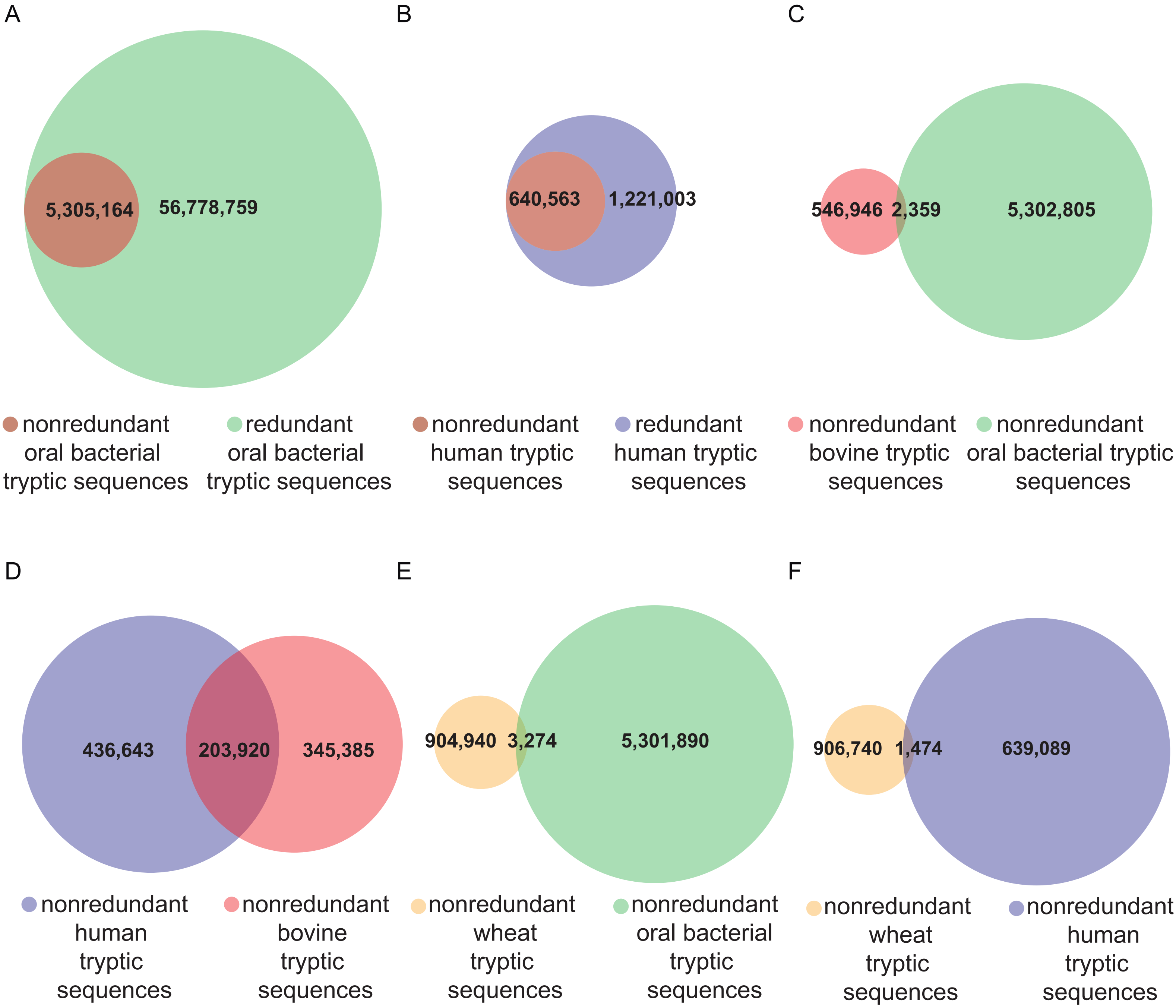

Figure S7: Overlap of tryptic sequences considered by MaxQuant for different organisms

The Venn Diagramms depict the number of tryptic peptides of amino acid lenght seven or greater for different organisms or groups of organisms like the oral microbiome. Only these peptides are considered in the search space by MaxQuant in our and the standard settings. (A) The oral microbiome contains multiple redundant tryptic sequences compared to humans (B) due to sequence identities across related taxa. Bovine and the oral microbiome (C), wheat and the oral microbiome (E) as well as wheat and humans (F) share few sequences in common. The respective human and bovine tryptic sequences show an overlap of 20% (D).

Figure S8

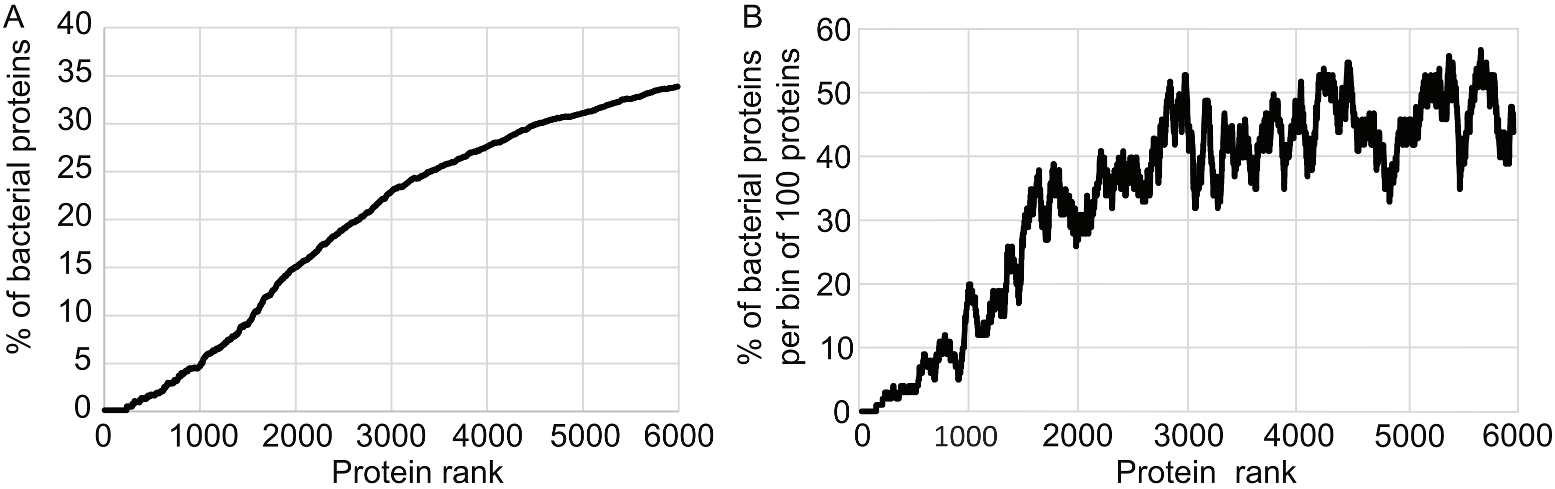

Figure S8: Distribution of bacterial proteins across the protein abundance range

(A) Percentage of bacterial proteins as a function of protein abundance rank. (B) Percentage of bacterial proteins per bin of 100 proteins.

Figure S9

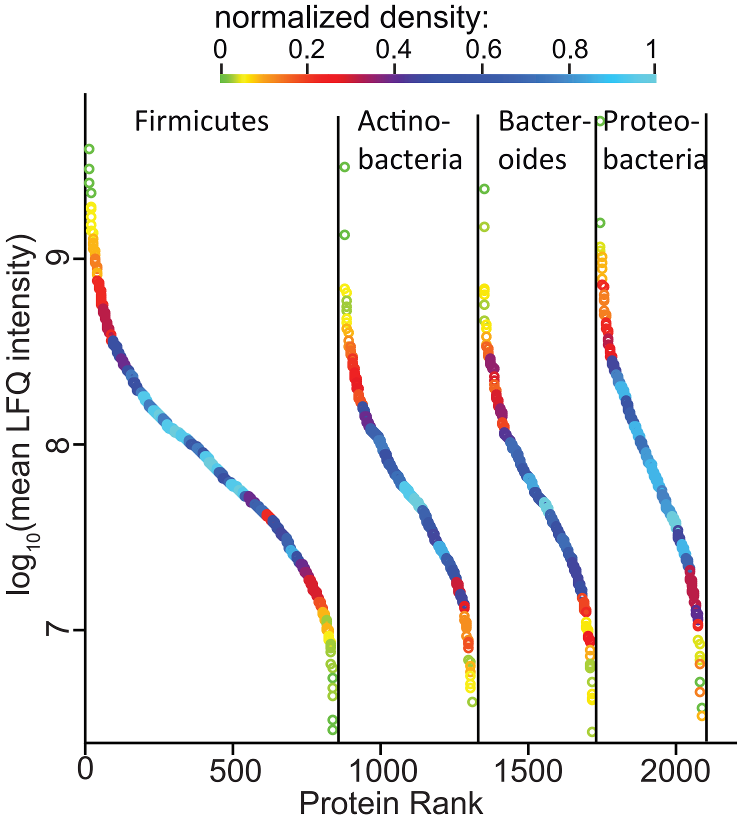

Figure S9: Dynamic range plot for the bacterial proteins stratified by phylum

Dynamic range plot of the bacterial proteins stratified by phylum for the four most abundant phyla.The protein density is color coded.
